# Neuro-computing solution for Lorenz differential equations through artificial neural networks integrated with PSO-NNA hybrid meta-heuristic algorithms: a comparative study

**DOI:** 10.1038/s41598-024-56995-2

**Published:** 2024-03-29

**Authors:** Muhammad Naeem Aslam, Muhammad Waheed Aslam, Muhammad Sarmad Arshad, Zeeshan Afzal, Murad Khan Hassani, Ahmed M. Zidan, Ali Akgül

**Affiliations:** 1 School of Mathematics, Minhaj University, Lahore, Pakistan; 2 Center for Mathematical Sciences (CMS), Pakistan Institute of Engineering and Applied Sciences, Nilore, Islamabad, 45650 Pakistan; 3 Department of Physics and Applied Mathematics (DPAM), Pakistan Institute of Engineering and Applied Sciences, Nilore, Islamabad, 45650 Pakistan; 4 Department of Physics, University of the Punjab, Lahore, Pakistan; 5 Department of Mathematics, Lahore Garrison University, Lahore, Pakistan; 6 Department of Mathematics, Ghazni University, Ghazni, Afghanistan; 7 Department of Mathematics, College of Science, King Khalid University, P.O. Box: 9004, 61413 Abha, Saudi Arabia; 8 Department of Computer Science and Mathematics, Lebanese American University, Beirut, Lebanon; 9 Department of Mathematics, Art and Science Faculty, Siirt University, 56100 Siirt, Turkey; 10 Department of Mathematics, Mathematics Research Center, Near East University, Near East Boulevard, 99138 Nicosia/Mersin 10, Turkey

**Keywords:** Artificial neural networks (ANN), Chaotic system, Particle swarm optimization (PSO), Neural network algorithm (NNA), Hybrid approach, Computational science, Computer science

## Abstract

In this article, examine the performance of a physics informed neural networks (PINN) intelligent approach for predicting the solution of non-linear Lorenz differential equations. The main focus resides in the realm of leveraging unsupervised machine learning for the prediction of the Lorenz differential equation associated particle swarm optimization (PSO) hybridization with the neural networks algorithm (NNA) as ANN-PSO-NNA. In particular embark on a comprehensive comparative analysis employing the Lorenz differential equation for proposed approach as test case. The nonlinear Lorenz differential equations stand as a quintessential chaotic system, widely utilized in scientific investigations and behavior of dynamics system. The validation of physics informed neural network (PINN) methodology expands to via multiple independent runs, allowing evaluating the performance of the proposed ANN-PSO-NNA algorithms. Additionally, explore into a comprehensive statistical analysis inclusive metrics including minimum (min), maximum (max), average, standard deviation (S.D) values, and mean squared error (MSE). This evaluation provides found observation into the adeptness of proposed AN-PSO-NNA hybridization approach across multiple runs, ultimately improving the understanding of its utility and efficiency.

## Introduction

Chaos theory is a branch of mathematics that studies the behavior of non-linear dynamic systems that are highly sensitive to initial conditions. This sensitivity leads to seemingly random behavior of the dynamics which is known as chaos. Due to this non-linearity the study of chaotic systems has been an active area of research in the fields of mathematics, physics, engineering, and other sciences. Many scientist and engineers have developed different techniques to analyzed and control chaotic dynamics, including bifurcation theory etc. These techniques have been applied to a wide range of systems, including electrical, mechanical electrical and biological. Overall, the study of chaotic system to be a very important and active area of research’s in different fields that has the potential to improve our understanding of the natural world and applications in different era.

These non-linear differential equations (DE) have become a standard model for chaotic systems. Kudryashov investigated analytical solutions to the Lorenz system that have been identified and has also categorized all precise solutions^1^. Bougoffa et al.^2^ studied the Lorenz system, which is known for representing the deterministic chaos in various practical fields such as laser phenomena, fluid mechanics, dynamos, thermosyphons, electric circuits, chemical processes, and forward osmosis. Algaba et al.^3^ constructed a model using Poincaré sections to analyze the worldwide connections formed by the subcritical T-point-Hopf bifurcation seen in the Lorenz system. In theoretical and numerical analysis of the traditional Lorenz model, Barrio and Serrano^4^ studied various different values of parameters and produced boundaries for the region where each positive semi-orbit converges to equilibrium point and established the constraints for the chaotic zone. The generalized Lorenz system has centers on center manifolds, as demonstrated by Algaba et al.^5^. For a continuous time nonlinear Lorenz chaotic system, Köse and Mühürcü^6^ developed controllers using the sliding mode control (SMC) and adaptive pole placement-based (APP) approaches^6^. Poland Studied the chemical model for the Lorenz equations^7^. All rational first integrals and Darboux polynomials of the Lorenz systems were described by Wu and Zhang^8^.

Algaba et al.^9^ computed a numerical analysis that made it possible to identify the bifurcations of the Lorenz system. Wu and Zhang^10^ investigated the extended Lorenz system subclass that has an invariant algebraic surface. Preturbulence in the three-dimensional phase space of the Lorenz system was demonstrated by Eusebius Doedel et al.^11^. Wu et al.^12^ examined the nonlinear chaotic dynamics model suing the reservoir computing (RC) to analyzed the behavior of the chaotic system. The dissipative Lorenz model has been analyzed by Sen and Tabor^13^ for the presence of symmetries. Chowdhury et al.^14^ applied the homotopy-perturbation method (HPM) for solution of Lorenz differential equation and compare with the 4th order Runge–Kutta (RK4) and multistage homotopy-perturbation method (MHPM). The simplified system of ordinary differential equations for heat flow in fluids, similar to the Lorenz system, models the changes in temperature and fluid movement within a system. Leonov et al.^15^ showed that the result for the Lyapunov dimension of the Lorenz system is valid for a wider range of system parameters. The Krishnan et al.^16^ used the laplace homotopy analysis method to compute the solution of Lorenz differential equations. Zlatanovska and Piperevski^17^ investigated the solution of the 3rd order shortened Lorenz system using integrability of a class of differential equations. Klöwer et al.^18^ focused on chaotic dynamical systems, non-periodic simulations using deterministic finite-precision numbers invariably result in orbits that eventually become periodic.

Artificial neural networks (ANN) have the ability to find approximate solutions of differential equations. Both Supervised and unsupervised neural networks used to find the solution of differential equation. A unsupervised neural networks integrated with optimization algorithm known as physics-informed generative adversarial networks (PI-GANs) has been proposed by yang et al.^19^ as a solution for solving differential equations. Raissi^20^ explored the use of deep learning techniques to address coupled forward–backward stochastic differential equations and the high-dimensional partial differential equations. Mattheakis et al.^21^ studied a physics-based unsupervised neural network (PI-NN) for solving differential equations (DEs) that depict the dynamic movement of systems. The PI-NN incorporates the Hamiltonian formulation through the use of a loss function, ensuring that the predicted solutions preserve energy. The loss function is only constructed using network predictions and does not require output data.

An unsupervised neural network was utilized by Mattheakis et al.^22^ to tackle a system of nonlinear differential equations. Raissi et al.^23^ introduced the concept of physics-informed neural networks, which are neural networks that are trained to solve nonlinear partial differential equations. Piscopo et al.^24^ used a specific approach to identify fully differentiable solutions for ordinary, coupled, and partial differential equations that have analytic solutions. They examined various network designs and found that even smaller networks produced exceptional results. Hagge et al.^25^ developed a system that model to solve differential equations. In their paper, Han et at.^26^ studied a deep learning-based approach that can tackle general high-dimensional partial differential equations (PDEs). The PDEs are rephrased using backward stochastic differential equations and the unknown solution's gradient is approximated by neural networks, similar to deep reinforcement learning where the gradient functions as the policy function. The effectiveness of artificial intelligence algorithms has been demonstrated by deterministic approaches, with particular emphasis on supervised learning and unsupervised artificial neural networks (ANN). These ANN have been exploited and refined using advanced stochastic techniques and swarm intelligence algorithms, leading to their successful application in different fields^27–29^.

In the field of nonlinear dynamics, the capabilities of these machine learning algorithms in solving challenging problems such as nonlinear oscillators and handling complex dynamics problems in different fields^30–40^. Furthermore, the versatility of these algorithms extends to solving nonlinear boundary value problems and other non-linear differential equations studied^41–55^ and show their effectiveness in different mathematical scenarios. Given the remarkable achievements in these applications, highly motivated to delve deeper into the potential of these unsupervised machine learning techniques to tackle Lorenz differential equations. Further, the prediction of proposed machine learning results compared with well-established (NDsolve) approach for validation.

## Non-linear Lorenz differential equations

The nonlinear chaotic system is a set of three non-linear differential equations that describe the behavior of a dynamical systems. The following system of three differential equations yields the Lorenz equations:

1 $$ \begin{gathered} \begin{array}{*{20}c} {\frac{dx\left( t \right)}{{dt}} = \sigma \left( {y\left( t \right) - x\left( t \right)} \right) } \\ {\begin{array}{*{20}c} {\frac{dy\left( t \right)}{{dt}} = Rx\left( t \right) - y\left( t \right) - x\left( t \right)z\left( t \right)} \\ {\frac{dz\left( t \right)}{{dt}} = x\left( t \right)y\left( t \right) - Bz\left( t \right) } \\ \end{array} } \\ \end{array} \hfill \\ t \in { }\left[ {0,{\text{ T}}} \right] \hfill \\ {\text{with initial conditions}} \hfill \\ x\left( 0 \right) = c_{1} , y\left( 0 \right) = c_{2} , z\left( 0 \right) = c_{3} \hfill \\ \end{gathered} $$

In this model three variables $x\left(t\right)$, $y\left(t\right)$ and $z\left(t\right)$ are be thought of as coordinates is 3-dimensional. The non-linear behavior of the chaotic system is examined by (3) three parameters $\sigma , R$, and $B$ which control the intensity of the non-linear interactions between the variables.

## Physics informed ANN based Lorenz differential equations

In recent years, there has been an advancing interest to use machine learning, deep learning approach to solve the chaotic systems. This algorithm, such as supervised neural networks (ANN) unsupervised neural networks (PINN) has been shown to be effective in approximating the numerical solutions to the equations. The physics informed neural network (PINN) based model is evolved to solve the chaotic system. Activation functions are a major component of neural networks (NN) and utilized for non-linearity into the ANN. There are different types of activation functions used in ANN such as sigmoid, ReLU, and tanh, each with their own properties and advantages. The choice of activation function can greatly impact the performance of ANN and accuracy of an ANN. The physics informed neural Lorenz Differential Equations are as follows:

2 $$ \left\{ {\begin{array}{*{20}c} {\hat{x}\left( t \right) = \mathop \sum \limits_{i = 1}^{k} a_{{x_{i} }} f\left( {w_{{x_{i} }} t + b_{{x_{i} }} } \right)} \\ {\frac{{d\hat{x}\left( t \right)}}{dt} = \mathop \sum \limits_{i = 1}^{k} a_{{x_{i} }} f{\prime} \left( {w_{{x_{i} }} t + b_{{x_{i} }} } \right)} \\ {\frac{{d^{2} \hat{x}\left( t \right)}}{{dt^{2} }} = \mathop \sum \limits_{i = 1}^{k} a_{{x_{i} }} f^{^{\prime\prime}} \left( {w_{{x_{i} }} t + b_{{x_{i} }} } \right)} \\ . \\ . \\ . \\ {\frac{{d^{m} \hat{x}\left( t \right)}}{{dt^{m} }} = \mathop \sum \limits_{i = 1}^{k} a_{{x_{i} }} f^{m} \left( {w_{{x_{i} }} t + b_{{x_{i} }} } \right)} \\ \end{array} } \right. $$

3 $$\left\{\begin{array}{c}\widehat{y}\left(t\right)=\sum_{i=1}^{k}{a}_{{y}_{i}}f({w}_{{y}_{i}}t+{b}_{{y}_{i}})\\ \frac{d\widehat{y}\left(t\right)}{dt}=\sum_{i=1}^{k}{a}_{{y}_{i}}f{^{\prime}}({w}_{{y}_{i}}t+{b}_{{y}_{i}})\\ \frac{{d}^{2}\widehat{y}\left(t\right)}{{dt}^{2}}=\sum_{i=1}^{k}{a}_{{y}_{i}}{f}{{^{\prime\prime}}}\left({w}_{{y}_{i}}t+{b}_{{y}_{i}}\right)\\ .\\ .\\ .\\ \frac{{d}^{m}y\left(t\right)}{{dt}^{m}}=\sum_{i=1}^{k}{a}_{{y}_{i}}{f}^{m}({w}_{{y}_{i}}t+{b}_{{y}_{i}})\end{array}\right.$$

4 $$\left\{\begin{array}{c}\widehat{z}\left(t\right)=\sum_{i=1}^{k}{a}_{{z}_{i}}f({w}_{{z}_{i}}t+{b}_{{z}_{i}})\\ \frac{dz\left(t\right)}{dt}=\sum_{i=1}^{k}{a}_{{z}_{i}}f{\prime}({w}_{{z}_{i}}t+{b}_{{z}_{i}})\\ \frac{{d}^{2}z\left(t\right)}{{dt}^{2}}=\sum_{i=1}^{k}{a}_{{z}_{i}}{f}^{{\prime}{\prime}}\left({w}_{{z}_{i}}t+{b}_{{z}_{i}}\right)\\ .\\ .\\ .\\ \frac{{d}^{m}\widehat{z}\left(t\right)}{{dt}^{m}}=\sum_{i=1}^{k}{a}_{{z}_{i}}{f}^{m}({w}_{{z}_{i}}t+{b}_{{z}_{i}})\end{array}\right.$$

where $\widehat{x}\left(t\right)=\frac{{a}_{{x}_{i}}}{1+{e}^{-({w}_{{x}_{i}}+{b}_{{x}_{i}})}}, \widehat{y}\left(t\right)=\frac{{a}_{{y}_{i}}}{1+{e}^{-({w}_{{y}_{i}}+{b}_{{y}_{i}})}} and \widehat{z}\left(t\right)=\frac{{a}_{{z}_{i}}}{1+{e}^{-({w}_{{z}_{i}}+{b}_{{z}_{i}})}}$ in sigmoid form in artificial neural networks (ANN) architecture.

The artificial neural networks (ANN) based derivatives of the Lorenz differential equations are given following:

5 $$ \hat{x}^{\prime}\left( t \right) = a_{{x_{ i} }} w_{{x _{i} }} \frac{{{\text{e}}^{{ - b_{{x _{i} }} - w_{{x _{i} }} t}} }}{{\left( {1 + {\text{e}}^{{ - b_{{x _{i} }} - w_{{x _{i} }} t}} } \right)^{2} }} $$

6 $$ \hat{y}^{\prime}\left( t \right) = a_{{y_{{{ }i}} }} w_{{y{ }_{i} }} \frac{{{\text{e}}^{{ - b_{{y{ }_{i} }} - w_{{y{ }_{i} }} t}} }}{{\left( {1 + {\text{e}}^{{ - b_{{y{ }_{i} }} - w_{{y{ }_{i} }} t}} } \right)^{2} }} $$

7 $$ \hat{z}^{\prime}\left( t \right) = a_{{z_{i} }} w_{{z _{i} }} \frac{{{\text{e}}^{{ - b_{{z _{i} }} - w_{{z _{i} }} t}} }}{{\left( {1 + {\text{e}}^{{ - b_{{z _{i} }} - w_{{z _{i} }} t}} } \right)^{2} }} $$

The artificial neural networks (ANNs) based Lorenz systems applied to solution taking ten (10) number of neuron in one hidden layer with ninety (90) correspondence weights $W=[{a}_{{x}_{i}}, {w}_{{x}_{i}},{b}_{{x}_{i}}, {a}_{{y}_{i}}, {w}_{{y}_{i}},{b}_{{y}_{i}}, {a}_{{z}_{i}}, {w}_{{z}_{i}},{b}_{{z}_{i}}$], $W$ are the weights and biases of unsupervised artificial neural networks (ANN) to optimize using hybrid PSO-NNA.

## ANN based fitness function

The ANN based fitness function is following

8 $${\upvarepsilon }_{x}=\sum_{j=1}^{k}{\left({a}_{{x}_{ i}}{w}_{{x }_{i}}\frac{{e}^{-{b}_{{x }_{i}}-{w}_{{x }_{i}}t}}{{\left(1+{e}^{-{b}_{{x }_{i}}-{w}_{{x }_{i}}t}\right)}^{2}}-\sigma \left(\frac{{a}_{{y}_{i}}}{1+{e}^{-({w}_{{y}_{i}}+{b}_{{y}_{i}})}}-\frac{{a}_{{x}_{i}}}{1+{e}^{-({w}_{{x}_{i}}+{b}_{{x}_{i}})}}\right)\right)}^{2}$$

9 $${\upvarepsilon }_{y}=\sum_{j=1}^{k}{\left({a}_{{y}_{ i}}{w}_{{y }_{i}}\frac{{{\text{e}}}^{-{b}_{{y }_{i}}-{w}_{{y }_{i}}t}}{{\left(1+{{\text{e}}}^{-{b}_{{y }_{i}}-{w}_{{y }_{i}}t}\right)}^{2}}-R\frac{{a}_{{x}_{i}}}{1+{e}^{-({w}_{{x}_{i}}+{b}_{{x}_{i}})}}+\frac{{a}_{{y}_{i}}}{1+{e}^{-({w}_{{y}_{i}}+{b}_{{y}_{i}})}}+\left(\frac{{a}_{{x}_{i}}}{1+{e}^{-({w}_{{x}_{i}}+{b}_{{x}_{i}})}}\right)\left(\frac{{a}_{{z}_{i}}}{1+{e}^{-({w}_{{z}_{i}}+{b}_{{z}_{i}})}}\right)\right)}^{2}$$

10 $${\upvarepsilon }_{z}=\sum_{j=1}^{k}{\left({a}_{{z}_{ i}}{w}_{{z }_{i}}\frac{{e}^{-{b}_{{z }_{i}}-{w}_{{z }_{i}}t}}{{\left(1+{e}^{-{b}_{{z }_{i}}-{w}_{{z }_{i}}t}\right)}^{2}}-\left(\frac{{a}_{{x}_{i}}}{1+{e}^{-({w}_{{x}_{i}}+{b}_{{x}_{i}})}}\right)\left(\frac{{a}_{{y}_{i}}}{1+{e}^{-({w}_{{y}_{i}}+{b}_{{y}_{i}})}}\right)-B\frac{{a}_{{z}_{i}}}{1+{e}^{-({w}_{{z}_{i}}+{b}_{{z}_{i}})}}\right)}^{2}$$

11 $${\upvarepsilon }_{ic}=\frac{1}{N}\sum_{j=1}^{k}\left({\left(\widehat{x}(t)-{\widehat{x}}_{0}\right)}^{2}+{\left(\widehat{y}(t)-{\widehat{y}}_{0}\right)}^{2}+{\left(\widehat{z}(t)-{z}_{0}\right)}^{2}\right)$$

12 $$\upvarepsilon ={\upvarepsilon }_{x}+{\upvarepsilon }_{y}+{\upvarepsilon }_{z}+{\upvarepsilon }_{ic}$$

where ${\widehat{x}}_{0}$, ${\widehat{y}}_{0}$ and ${z}_{0}$ initial conditions, $\upvarepsilon$ is fitness function based on physics informed neural networks and $N$ is total number of initial conditions.

## Meta-heuristic optimization algorithms

Meta-heuristic algorithms have used a pivotal role in tackling a wide range of non-linear optimization problems both constrained and unconstrained, across various engineering domains. These optimization algorithms are designed to find approximate solutions of the problems when traditional optimization techniques struggle due to the complexity or non-linearity of the objective functions and constraints involved. Over the years, researchers have developed numerous meta-heuristics each with its unique approach, methodology and strengths. Notable algorithms that have made significant contributions to the engineering field include Genetic Algorithm, Particle Swarm, Firefly Algorithm, Water Cycle, Ant Colony, Levy Flight, Artificial Bee Colony, Hunting Search, Simulated Annealing, and many others.

### Particle swarm optimization (PSO)

Particle Swarm Optimization (PSO) stands as a remarkable evolutionary optimization algorithm inspired by specifically the behavior of birds within a swarm. Its main objective is to analyze complex non-linear complex optimization problems that difficult to solve using traditional approaches. PSO helps to alter particle placements based on swarm-discovered global best solutions as well as individual best through a iterative phases. PSO functions by sustaining a population of particles, each of which represents a potential solution to the optimization issue, much like a flock of birds adjusting to their environment. The best-performing location for each particle, called the personal best (pbest), and the best-performing position for the entire swarm, called the global best (gbest), control how the particles moves to optimal position. Particles collaborate indirectly by continually fine-tuning their locations in relation to these standards, therefore traversing the optimization terrain with effectiveness.

The use of PSO in several domain demonstrates its adaptability and helpful. Notably, PSO has shown useful in the fields of robotics and wireless networks, where it has optimized resource allocation and network parameter setup^56,57^. In the context of power systems, PSO has played a crucial role in optimizing energy distribution and load management^58,59^. In complex scheduling problems like job shop scheduling, where tasks must be allocated efficiently among limited resources, PSO has showcased its prowess^60,61^. This balance ensures that the algorithm not only explores the solution space thoroughly but also exploits the promising areas identified during the exploration. This trait is particularly valuable when dealing with multifaceted optimization scenarios, where the landscape can be rugged and intricate. Researchers utilized PSO in different complex non-linear problems^64–73^. Starting with randomly chosen particles each iteration updates particle positions and velocities based on their latest best local position ${P}_{LB}^{x-1}$ and global position ${P}_{GB}^{x-1} .$

The continuous standard PSO framework involves updating particle positions and velocities using the following general formulas: particle velocity update:

13 $${v}_{i}^{x}=w{v}_{i}^{t-1}+{c}_{1}{r}_{1}\left({P}_{LB}^{x-1}-{X}_{i}^{x-1}\right)+{c}_{2}{r}_{2}({P}_{GB}^{x-1}-{X}_{i}^{x-1})$$

Particle position update

14 $${X}_{i}^{x}={X}_{i}^{x-1}+{v}_{i}^{x-1}$$

In these equations $i$ ranges from 1 to $p$ where $p$ is the total number of particles. Where ${X}_{i}$ represents the position of the $ith$ particle in the swarm, while ${v}_{i}$ is its velocity vector. The framework incorporates parameters such as $V$ (inertia weight), ${c}_{1}$ and ${c}_{2}$ (local and global social acceleration constants), and a weight that linearly decreases from $[0 , 1]$ Random vectors ${r}_{1}$ and ${r}_{2}$ are constrained between $[0 , 1]$.

### Neural network algorithm (NNA)

Introducing a novel variant of the NNA^74^ this distinctive evolutionary approach draws inspiration from both biological nervous systems and ANN. While ANN prediction purposes, the NNA ingeniously amalgamates neural network principles with randomness to tackle optimization problems. By using the intrinsic structure of neural networks NNA demonstrates strong global optimization search performance. Remarkably, NNA sets itself apart from traditional meta-heuristic methods by relying solely on population size and stopping criteria, eliminating the need for additional parameters^74^. NNA is a population-centered optimization algorithm that consists of the following four key elements:

#### Update population

By NNA the vector for population ${X}_{t}=\{{x}_{1}^{t},{ x}_{2}^{t}, {x}_{3}^{t},\dots ,{x}_{M}^{t}\}$ undergoes updates through the weight matrix ${W}^{t}=\{{w}_{1}^{t},{ w}_{2}^{t}, {w}_{3}^{t},\dots ,{w}_{M}^{t}\}$ where ${w}_{t}^{i}=\{{w}_{i,1}^{t}, {w}_{i,2}^{t}, {w}_{i,3}^{t},\dots , {w}_{i,M}^{t}\}$ represents the weight vector of the ith individual, and ${x}_{t}^{i}=\{{x}_{i,1}^{t}, {x}_{i,2}^{t}, {x}_{i,3}^{t},\dots , {x}_{i,D}^{t}\}$ signifies the position of the *i*th individual. Notably, D denotes the count of variables. In addition, the process of creating a new population may be expressed numerically as follows:

15 $${x}_{new,i}^{t}=\sum_{i=1}^{Q}{w}_{i,k}^{t}\times {x}_{i}^{t}, i={1,2},3,\dots ,Q, k={1,2},3,\dots ,Q$$

16 $${x}_{i}^{t}={x}_{i}^{t}+{x}_{new,i}^{t}, i={1,2},3,\dots ,Q$$

Here Q denotes the size of the population, and t is the number of iterations that are currently in use. ${x}_{new,i}^{t}$ Represents the solution for the *i*th person at the same time point, computed with the proper weights, and ${x}_{i}^{t}$, represents the solution for the *i*th individual at time t. Moreover, the following formulation applies to the weight vector ${w}_{i}^{t}$:

17 $$\sum_{i=1}^{Q}{w}_{i,k}^{t}=1, 0<{w}_{i,k}^{t}<1, i={1,2},3,\dots ,Q, k={1,2},3,\dots ,Q$$

#### Update weight matrix

As depicted in Eq. (35) the weight matrix ${W}^{t}$ assumes a pivotal role within NNA process of generating a novel population. The dynamics of the weight matrix ${W}^{t}$ can be refined through:

18 $${w}_{i}^{t}=\left|{w}_{i}^{t}+2\times {\lambda }_{2}({w}_{obj}^{t}-{w}_{i}^{t})\right|, i={1,2},3,\dots ,Q$$

where ${\lambda }_{2}$ randomly value from [0, 1] and ${w}_{obj}^{t}$ is the objective/fitness weight vector. It’s highlight that both the objective weight vector ${w}_{obj}^{t}$ and the target value ${x}_{obj}^{t}$ share corresponding indices. To elaborate further, if ${x}_{obj}^{t}$ matches ${x}_{v}^{t}$, ($v \in [1,\text{ Q}]$) at $t$ where $t$ is time, then ${w}_{obj}^{t}$ is equivalently to ${w}_{v}^{t}$.

#### Bias operator

The bias operator in NNA is to increase the organization ability to explore the best optimal values. A modification factor, represented as ${\beta }_{1}$, becomes important for determining the amount of bias that has been introduced. Updates to this factor can be obtained through:

19 $${\beta }_{1}^{t+1}=0.99{\beta }_{1}^{t}$$

The bias operator consists of a bias weight matrix and a bias population, which are each described as follows: two variables are involved in the bias population operator: a set designated as P and a randomly generated number ${Q}_{p}$. The lower and higher bounds of the variables are represented by the expressions $l=({l}_{1}, {l}_{2}, {l}_{3}, \dots ,{l}_{D})$ and $u=({u}_{1}, {u}_{2}, {u}_{3}, \dots ,{u}_{D})$, respectively. ${\beta }_{1}^{t}\times D$, which represents the ceiling value of the product of ${\beta }_{1}^{t}$ and $D$, is used to calculate ${M}_{p}$. ${Q}_{p}$ randomly chosen numbers from the interval $[0,D]$ make up the set P. As a result, the bias population is exactly defined as follows:

20 $${x}_{i,P(S)}^{t}={l}_{P(S)}^{t}+\left({u}_{P\left(S\right)}-{l}_{P\left(S\right)}\right)\times {\lambda }_{3}, S={1,2},3,\dots ,{Q}_{P}$$

${\lambda }_{3}$ stands for a uniformly distributed random number inside the interval $[0, 1].$ Two more variables are included in the bias matrix: a set designated as R and a randomly generated integer ${Q}_{w}$. The ceiling of $[{\beta }_{1}^{t}\times Q]$ is used to compute the value of ${Q}_{w}$. Parallel to this, the set $R$ is made up of ${Q}_{w}$ integers that are chosen at random from the interval [0, Q]. As a result, the bias weight matrix may be precisely identified as

21 $${w}_{i,R(r)}^{t}={\lambda }_{4}, r={1,2},3,\dots , {Q}_{w}$$

where ${\lambda }_{4},$ is a random number between $[0, 1]$ subject to uniform distribution.

#### Transfer operator (TO)

In order to reach the present optimal solution, the transfer operator must produce the best solution possible with an emphasis on NNA local search using the following equation

22 $${x}_{i}^{t+1}={x}_{i}^{t}+2{\lambda }_{5}\left({x}_{obj}^{t}-{x}_{i}^{t}\right), i={1,2},3,\dots ,Q$$

where ${\lambda }_{5}$ a random value from 0 to 1. The NNA initialized following equation

23 $${x}_{i,k}^{t}={l}_{k}+\left({u}_{k}-{l}_{k}\right)\times {\lambda }_{6}, i={1,2},3,\dots , Q, k={1,2},3,\dots ,D$$

where ${\lambda }_{6}$ is random value from $[0, 1]$

**Figure:**
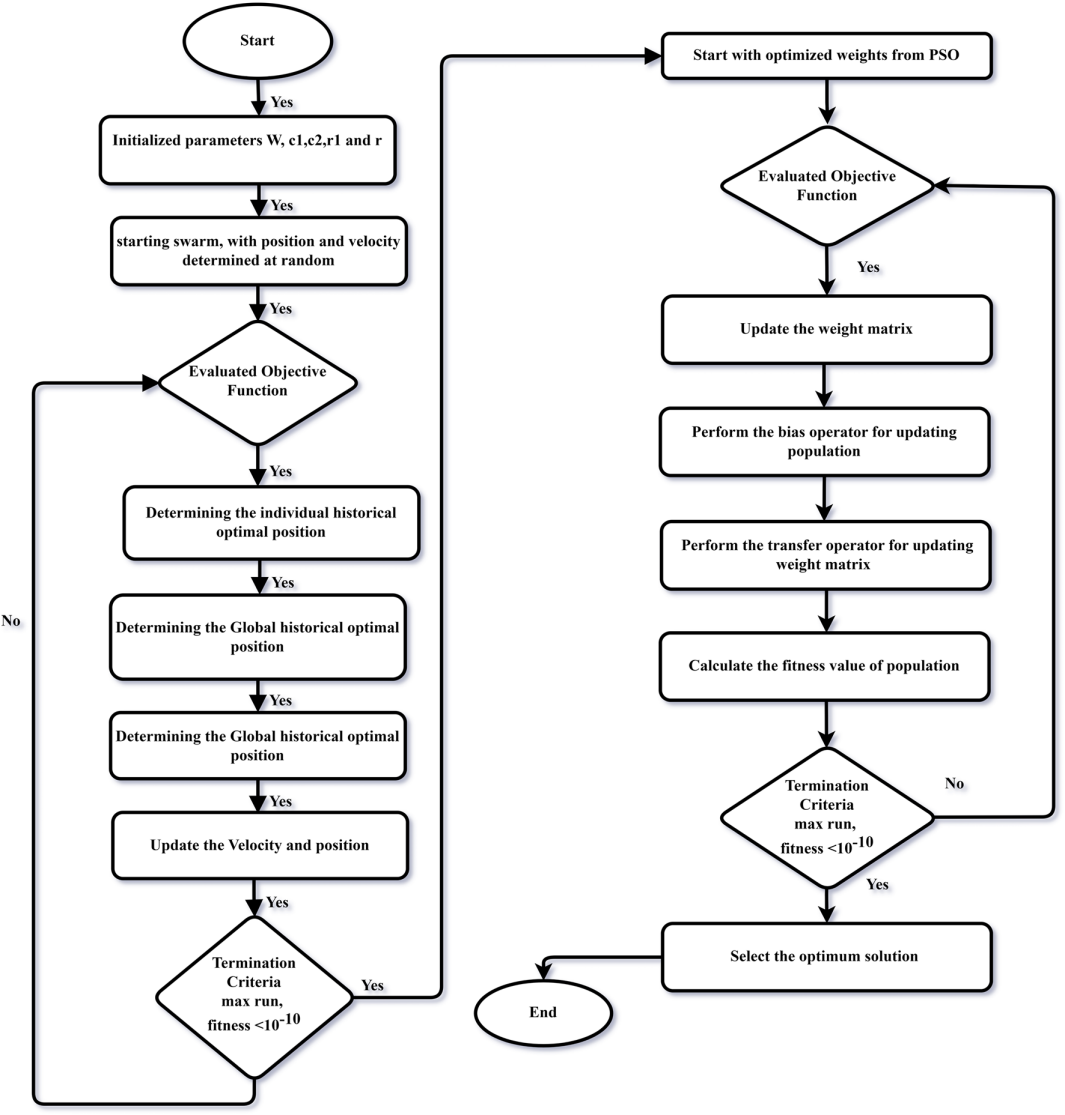
Flow Chart: Generic flow chart of PSO-NNA

## Results and discussion

### Case 1

In this case, Lorenz differential equations in given equation has been evaluated by taking the fixed numerical values of the parameters σ, R and $B$. The ANN based fitness function of Lorenz differential equation for this case written as,

24 $$\left\{\begin{array}{c}{\upvarepsilon }_{x}=\sum_{i=1}^{11}{\left(\frac{d\widehat{x}(t)}{dt}-0.1\left(\widehat{y}(t)-\widehat{x}(t)\right)\right)}^{2} \\ {\upvarepsilon }_{y}=\sum_{i=1}^{11}{\left(\begin{array}{c}\frac{d\widehat{y}\left(t\right)}{dt}-0.2\widehat{x}\left(t\right)+\widehat{y}\left(t\right)\\ +\widehat{x}(t)\widehat{z}(t)\end{array}\right)}^{2}\\ {\upvarepsilon }_{z}=\sum_{i=1}^{11}{\left(\frac{d\widehat{z}(t)}{dt}-\widehat{x}(t)\widehat{y}(t)-0.3\widehat{z}(t) \right)}^{2} \\ {\upvarepsilon }_{ic}=\frac{1}{3}\sum_{j=1}^{11}\left(\begin{array}{c}{\left(\widehat{x}\left(0\right)-0\right)}^{2}+{\left(\widehat{y}\left(0\right)-1\right)}^{2}\\ +{\left(\widehat{z}(0)-0\right)}^{2}\end{array}\right)\end{array}\right.$$

The artificial neural networks (ANNs) scheme are applied to solution of the problem taking ten (10) number of neuron in hidden layer with ninety (90) correspondence weights $W=[{a}_{{x}_{i}}, {w}_{{x}_{i}},{b}_{{x}_{i}}, {a}_{{y}_{i}}, {w}_{{y}_{i}},{b}_{{y}_{i}}, {a}_{{z}_{i}}, {w}_{{z}_{i}},{b}_{{z}_{i}}$], the fitness function constructed using artificial neural network for this case taking $t \in [{0,1}]$ with step size $0.1$. To find the optimal weights of the artificial neural networks used hybridization of particle swarm optimization (PSO) and neural network algorithm (NNA).

25 $$\upvarepsilon =\left({\upvarepsilon }_{x}+{\upvarepsilon }_{y}+{\upvarepsilon }_{z}+{\upvarepsilon }_{ic}\right)$$

The fitness function shown Eq. (25) with 90 weights and biases, denoted by set $W$, here $W=[{a}_{{x}_{i}}, {w}_{{x}_{i}},{b}_{{x}_{i}}, {a}_{{y}_{i}}, {w}_{{y}_{i}},{b}_{{y}_{i}}, {a}_{{z}_{i}}, {w}_{{z}_{i}},{b}_{{z}_{i}}$], as well as its components are

$${a}_{{x}_{i}}=\{{a}_{{x}_{1}},{a}_{{x}_{2}}, {a}_{{x}_{3}},\dots ,{a}_{{x}_{k}}\}$$

$${w}_{{x}_{i}}=\{{w}_{{x}_{1}},{w}_{{x}_{2}}, {w}_{{x}_{3}},\dots ,{w}_{{x}_{k}}\}$$

$${b}_{{x}_{i}}=\{{b}_{{x}_{1}},{b}_{{x}_{2}}, {b}_{{x}_{3}},\dots ,{b}_{{x}_{k}}\}$$

$${a}_{{y}_{i}}=\{{a}_{{y}_{1}},{a}_{{y}_{2}}, {a}_{{y}_{3}},\dots ,{a}_{{y}_{k}}\}$$

$${w}_{{y}_{i}}=\left\{{w}_{{y}_{1}},{w}_{{y}_{2}}, {w}_{{y}_{3}},\dots ,{w}_{{y}_{k}}\right\}$$

$${b}_{{y}_{i}}=\left\{{b}_{{y}_{1}},{b}_{{y}_{2}}, {b}_{{y}_{3}},\dots ,{b}_{{y}_{k}}\right\}$$

$${a}_{{z}_{i}}=\{{a}_{{z}_{1}},{a}_{{z}_{2}}, {a}_{{z}_{3}},\dots ,{a}_{{z}_{k}}\}$$

$${w}_{{z}_{i}}=\{{w}_{{z}_{1}},{w}_{{z}_{2}}, {w}_{{z}_{3}},\dots ,{w}_{{z}_{k}}\}$$

$${b}_{{z}_{i}}=\{{b}_{{z}_{1}},{b}_{{z}_{2}}, {b}_{{z}_{3}},\dots ,{b}_{{z}_{k}}\}$$

In this research, introduced a novel hybridization approach that combines unsupervised artificial neural networks (ANN) with two powerful global optimization algorithms: Particle Swarm Optimization (PSO) and Neural Network Algorithm (NNA) as (ANN-PSO-NNA). This hybrid approach to aims to find the optimum weights and biases for ANN based of Lorenz differential equations. Our ANN-PSO-NNA methodology involves a two-step process. Firstly, PSO employ to generate randomized weight sets, which serves as a promising initialization point. Further employ NNA to more fine-tune and optimize these weight sets for more accurate results. This hybrid strategy showcases the potential for achieving superior results in optimizing ANN based differential equation within defined initial conditions constraints.
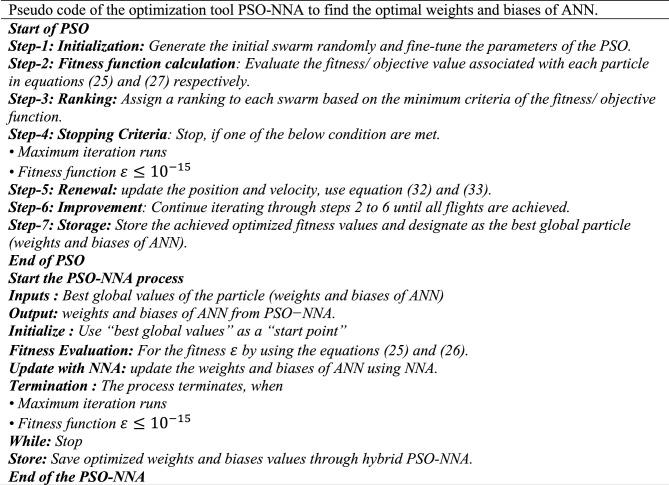


**Figure 1:**
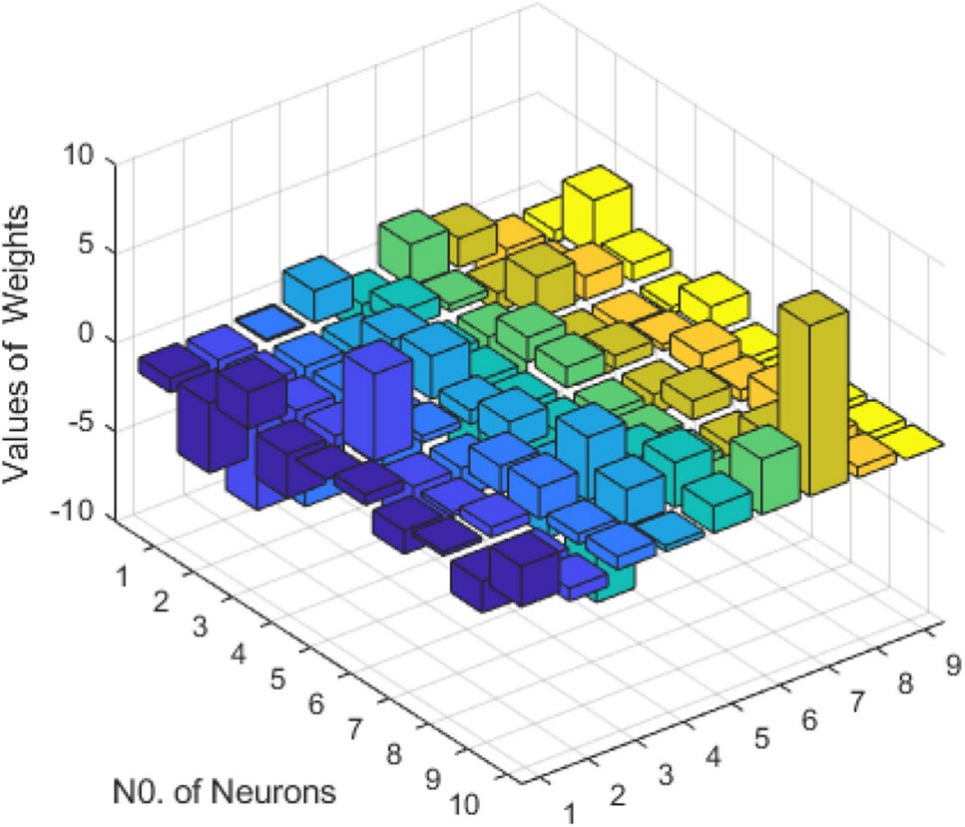
Optimized weights through ANN-PSO-NNA for case 1.

**Figure 2:**
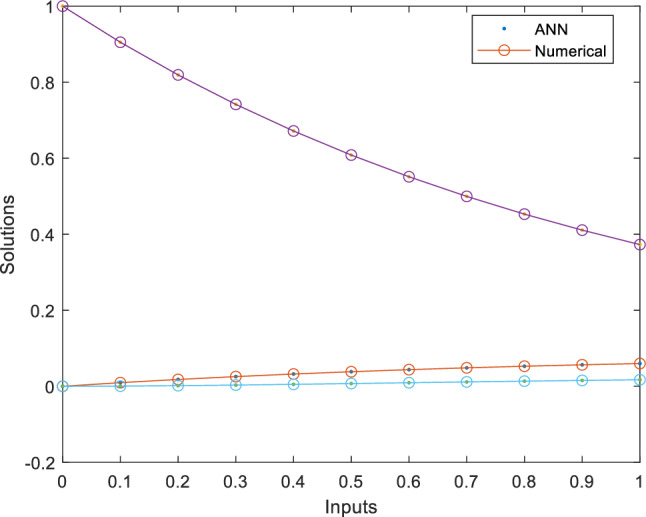
Comparison solution of Lorenz differential equation using NDsolve and ANN-PSO-NNA for case 1.

**Figure 3:**
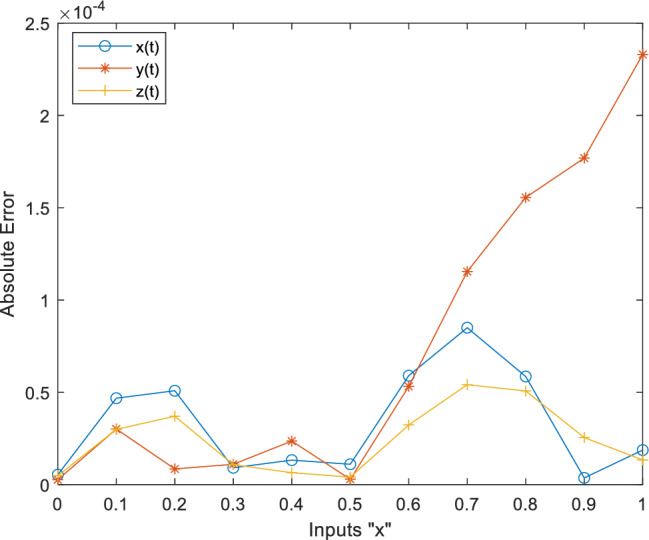
Absolute error between ANN-PSO-NNA and NDsolve for case 1.

**Figure 4:**
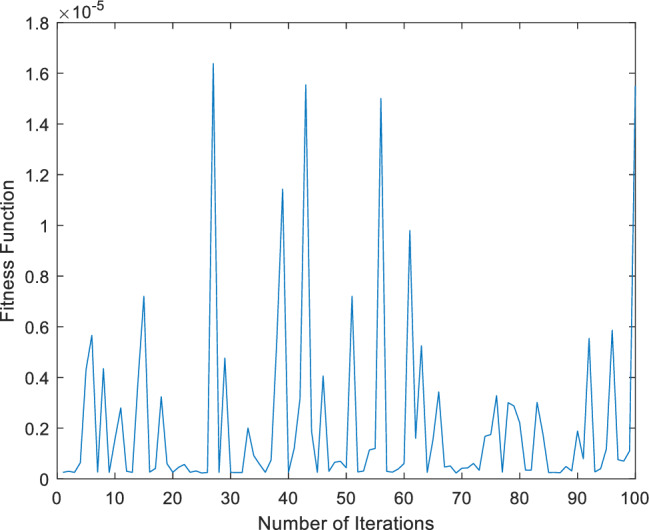
Fitness function across one hundred (100) independence runs for case 1.

This fitness function represented in Eq. (25) is meticulously designed to converge towards zero using these optimized set of weights and biases. The resulting optimal weight set, denoted as $W$ and meticulously tabulated in Tables 1, 2 and 3 and plotted in Fig. 1. Delving into the realm involves exploring of ANN-PSO-NNA to calculate and predict the ANN based $\widehat{x}\left(t\right), \widehat{y}\left(t\right)$ and $\widehat{z}\left(t\right)$. These predicted values are then meticulously tabulated in Tables 4, 5 and 6. Our study takes a step further by conducting a comprehensive comparison analysis, pitting the solutions from the NDsolve method against those generated by our innovative numerical ANN-PSO-NNA hybrid algorithm represented in Fig. 2. The error metric especially absolute errors (AE) inherent in these predictions are tabulated in Tables 4, 5 and 6, graphical visually in Fig. 3. The crux of proposed machine learning evaluation lies in the accuracy and convergence evolution of the ANN-PSO-NNA. To ensure robustness, the proposed ANN-PSO-NNA approach undergoes rigorous testing across a (100) hundred independent runs. The fitness function for the Lorenz differential equation is established by this procedure for two (2) separate scenarios. The effectiveness of obtaining precise and convergent presented in Fig. 4 for case 1 is assessed by using the fitness function with ANN-PSO-NNA across one hundred (100) independent runs.

**Table 1:** The best optimized set of weighs through ANN-PSO-NNA for x (t) for with ${\varvec{\sigma}}$ = 0.1, R = 0.2 and B = 0.3.

${w}_{{x}_{i}}$	${b}_{{x}_{i}}$	${a}_{{x}_{i}}$
− 0.813325309	0.124153837	− 1.977661201
− 3.905335359	− 7.842802359	− 7.010510834
2.070647136	− 1.13823337	− 1.072293243
− 2.459776325	− 2.87089813	− 0.653293577
0.020306929	0.390174591	4.893449037
0.59295416	− 1.651579931	− 3.117918392
− 1.311168679	1.540327448	− 0.272077258
0.239241627	1.778650834	0.575686377
− 1.76626202	0.591203789	− 1.30910321
2.260099588	0.751550886	− 0.727266222

**Table 2:** The best optimized set of weighs through ANN-PSO-NNA for y (t) for with ${\varvec{\sigma}}$ = 0.1, R = 0.2 and B = 0.3.

${w}_{{y}_{i}}$	${b}_{{y}_{i}}$	${a}_{{y}_{i}}$
1.79513648	− 1.619284162	2.33332237
− 3.479169522	1.049353281	0.391080791
1.901550548	− 1.868873265	− 2.946745976
2.405750129	− 5.427098595	1.388433611
0.791306332	− 2.256709224	1.136508252
1.568062177	− 3.343812861	− 1.295923739
− 3.497286829	− 8.177244791	− 0.800059922
3.55394297	1.339271967	− 1.199927409
2.043766309	2.527430403	− 1.014961309
0.322003823	1.493407826	3.11031704

**Table 3:** The best optimized set of weighs through ANN-PSO-NNA for z (t) for with ${\varvec{\sigma}}$ = 0.1, R = 0.2 and B = 0.3.

${w}_{{z}_{i}}$	${b}_{{z}_{i}}$	${a}_{{z}_{i}}$
1.640553936	− 2.655547759	− 0.646854678
− 1.030352672	− 2.065588875	3.135717185
2.323983541	1.302367848	0.998378559
− 2.313212076	− 0.642094131	− 0.391409694
0.750563972	0.343277015	1.435755696
− 1.072402348	1.233927562	− 0.587671128
1.077467937	0.639052951	− 0.168720266
− 0.725073923	1.769849572	− 0.426233397
2.009219372	0.773859075	− 1.259451854
9.576093207	0.596666302	− 0.013176696

**Table 4:** An evaluation of the accuracy of x (t) using NDsolve and ANN-PSO-NNA for case 1, with ${\varvec{\sigma}}$ = 0.1, ${\varvec{R}}=0.2$ and ${\varvec{B}}=0.3.$

t	${x(t)}_{Numerical}$	${\widehat{x}(t)}_{ANN}$	${AE(x(t))}_{ANN}$
0	0	5.40E−06	5.4046E−06
0.1	0.009468358	0.009515209	4.6851E−05
0.2	0.017943263	0.017994147	5.0885E−05
0.3	0.025521751	0.025530937	9.1863E−06
0.4	0.0322914	0.032278219	1.3181E−05
0.5	0.038331266	0.038342661	1.1395E−05
0.6	0.043712682	0.043772499	5.9818E−05
0.7	0.048500038	0.048586562	8.6524E−05
0.8	0.052751446	0.052812704	6.1258E−05
0.9	0.056519362	0.056520039	6.7777E−07
1	0.05985115	0.059839029	1.212E−05

**Table 5:** An evaluation of the accuracy of y (t) using NDsolve and ANN-PSO-NNA for case 1, with ${\varvec{\sigma}}$ = 0.1, R = 0.2 and B = 0.3.

t	${x(t)}_{Numerical}$	${\widehat{x}(t)}_{ANN}$	${AE(y(t))}_{ANN}$
0	1	0.999997313	2.69E−06
0.1	0.904930603	0.904960789	3.02E−05
0.2	0.819077383	0.819086889	9.51E−06
0.3	0.741542942	0.741558898	1.60E−05
0.4	0.671516632	0.67155452	3.79E−05
0.5	0.608266533	0.608300919	3.44E−05
0.6	0.551132369	0.551137058	4.69E−06
0.7	0.499518461	0.499497847	2.06E−05
0.8	0.452887662	0.452874396	1.33E−05
0.9	0.410755511	0.410778667	2.32E−05
1	0.372685019	0.372719378	3.44E−05

**Table 6:** An evaluation of the accuracy of z (t) using NDsolve and ANN-PSO-NNA for case 1, with ${\varvec{\sigma}}$ = 0.1, R = 0.2 and B = 0.3.

t	${x(t)}_{Numerical}$	${\widehat{x}(t)}_{ANN}$	${AE(z(t))}_{ANN}$
0	0	− 4.85E−06	4.8524E−06
0.1	0.000446736	0.000476715	2.99791E−05
0.2	0.001598645	0.001635725	3.70799E−05
0.3	0.003222261	0.003233088	1.08268E−05
0.4	0.0051386	0.005132109	6.49118E−06
0.5	0.007211748	0.007215832	4.08402E−06
0.6	0.009340018	0.009372364	3.2346E−05
0.7	0.011448536	0.011502689	5.41535E−05
0.8	0.013483495	0.013534246	5.07514E−05
0.9	0.015407483	0.015433005	2.55217E−05
1	0.017195735	0.017209134	1.33988E−05

**Figure 5:**
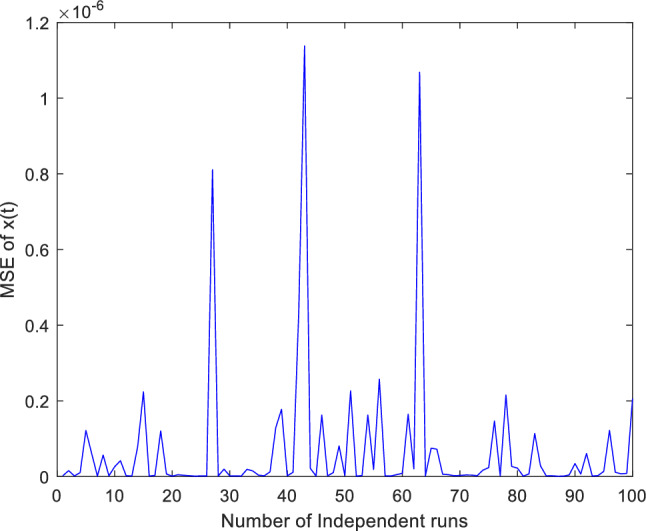
Mean square error (MSE) plotted over 100 independent runs of ${\varvec{x}}({\varvec{t}})$ for case 1.

**Figure 6:**
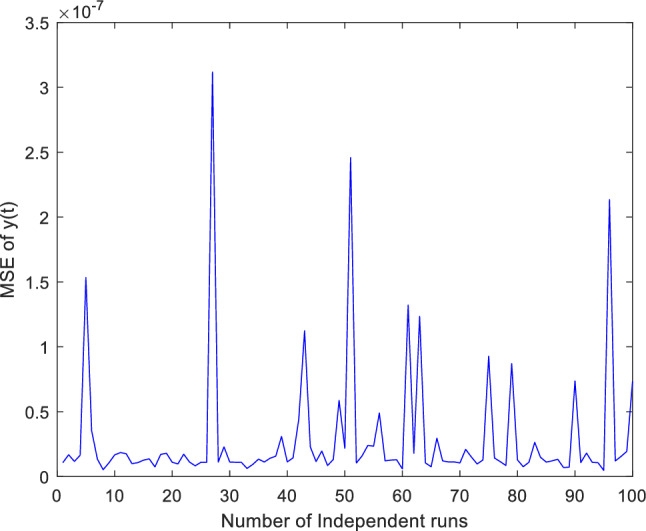
Mean square error (MSE) plotted over 100 independent runs of ${\varvec{y}}({\varvec{t}})$ for case 1.

**Figure 7:**
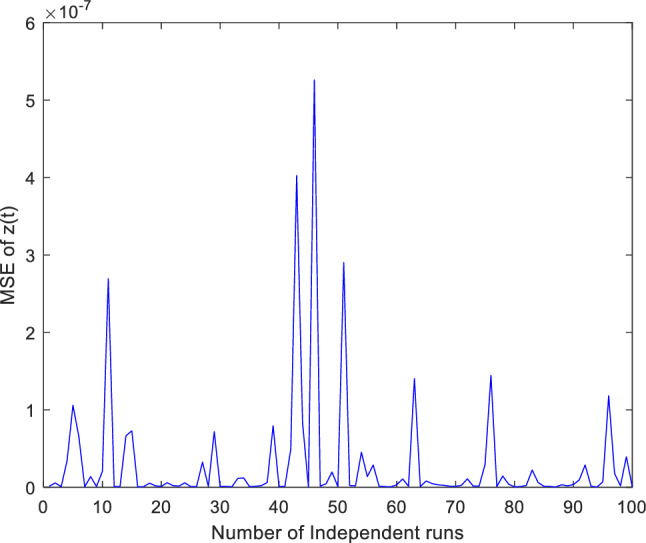
Mean square error (MSE) plotted over 100 independent runs of ${\varvec{z}}({\varvec{t}})$ for case 1.

Additionally, concentrate on validation the proposed ANN-PSO-NNA approach performance by thoroughly investigation its convergence behavior. To assess its efficacy computed numerically mean square error (MSE) over a (100) one hundred independent runs. The acquired numerical MSE values offer insightful analysis to verify and efficiency the proposed hybrid ANN-PSO-NNA capacity to converge towards optimal solutions. Plots illustrate the convergence patterns of the proposed ANN-PSO-NNA hybrid algorithm are used to show MSE over (100) separate runs. Figures 5, 6 and 7 display the plotted MSE values for each of the case 1.

### Case 2

In the context of this study, delve into the analysis of the Lorenz differential equation problem using the machine learning techniques. The focus of our investigation lies in evaluating the behavior of the equation under specific conditions. For case (2) a comprehensive evolution selected fixed values for the parameters $\sigma , R$ and $B$ which play a important role in shaping the dynamics of the Lorenz equation.

26 $$\left\{\begin{array}{c}{\upvarepsilon }_{x}=\sum_{i=1}^{11}{\left(\frac{d\widehat{x}(t)}{dt}-1\left(\widehat{y}(t)-\widehat{x}(t)\right)\right)}^{2} \\ {\upvarepsilon }_{y}=\sum_{i=1}^{11}{\left(\begin{array}{c}\frac{d\widehat{y}\left(t\right)}{dt}-2\widehat{x}\left(t\right)+\widehat{y}\left(t\right)\\ +\widehat{x}(t)\widehat{z}(t)\end{array}\right)}^{2}\\ {\upvarepsilon }_{z}=\sum_{i=1}^{11}{\left(\frac{d\widehat{z}(t)}{dt}-\widehat{x}(t)\widehat{y}(t)-3\widehat{z}(t) \right)}^{2} \\ {\upvarepsilon }_{ic}=\frac{1}{3}\sum_{j=1}^{11}\left(\begin{array}{c}{\left(\widehat{x}\left(0\right)-0\right)}^{2}+{\left(\widehat{y}\left(0\right)-1\right)}^{2}\\ +{\left(\widehat{z}(0)-0\right)}^{2}\end{array}\right)\end{array}\right.$$

27 $$\upvarepsilon =\left({\upvarepsilon }_{x}+{\upvarepsilon }_{y}+{\upvarepsilon }_{z}+{\upvarepsilon }_{ic}\right)$$

**Figure 8:**
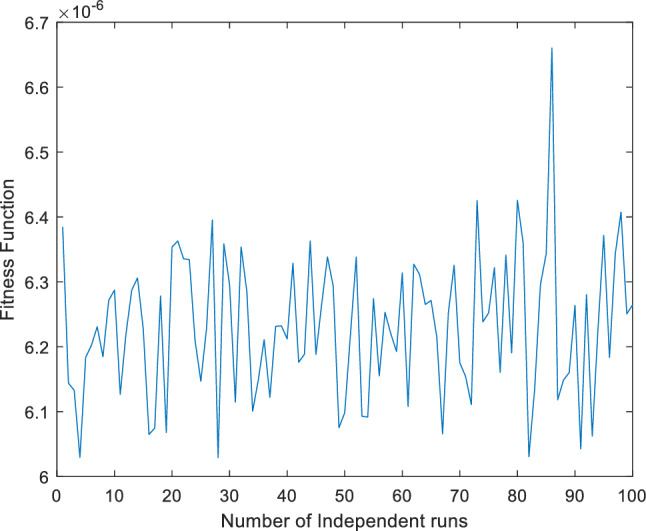
Fitness function across one hundred (100) independence runs for case 2.

**Figure 9:**
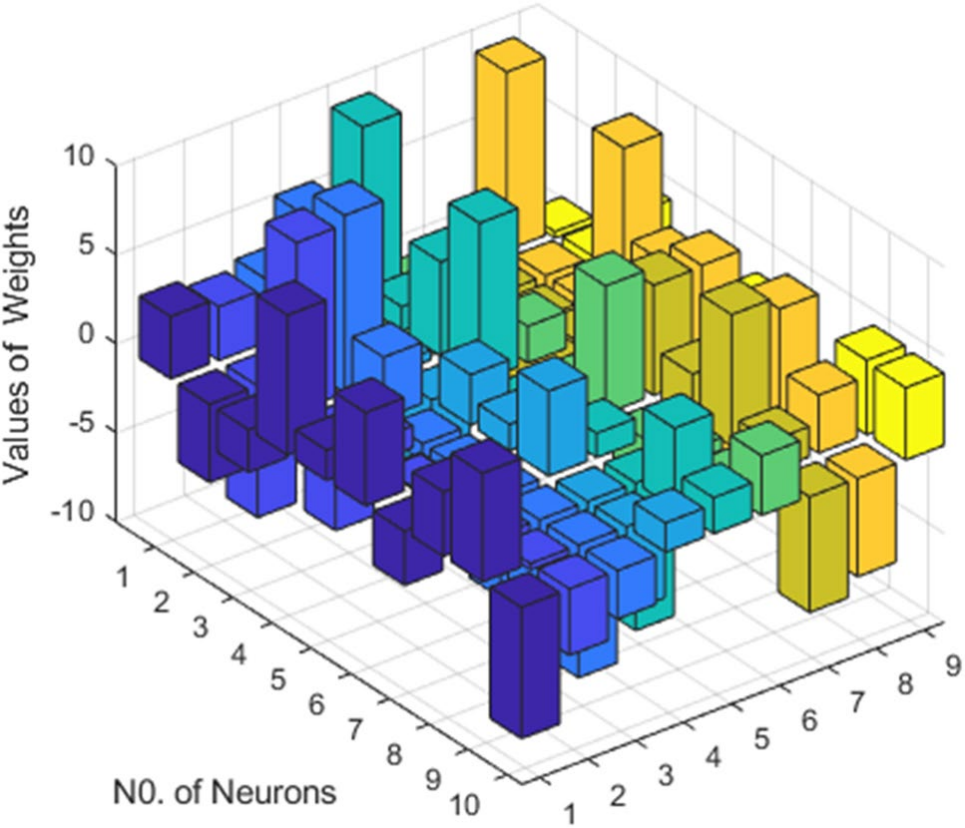
Optimized weights through ANN-PSO-NNA for case 2.

In this article introduce a novel hybridization approach for case (2) that combines physics informed neural networks (PINN) with two powerful global optimization algorithms: Particle PSO and NNA called ANN-PSO-NNA. This hybrid machine learning approach to aims to find the optimum weights and biases for ANN based of chaotic behavior of Lorenz differential equations. Machine learning approach ANN-PSO-NNA involves a 2-step process. Firstly PSO used to optimal to generate randomized weight and biases of ANN sets, which serves as a promising initialization for NNA. Further, NNA employ to more fine-tune and optimize these set $W$. This hybridization strategy showcases the potential for achieving more superior numerical results in optimizing ANN based differential equation within associated initial conditions. The fitness / objective function as represented for case (2) in Eq. (27), has been meticulously crafted to steadily converge towards zero (0) for (100) independent runs illustrated in Fig. 8. This is achieved by leveraging the best set of weights and biases of ANN obtained through the PSO-NNA hybridization approach. The resulting set of optimal weights, denoted as W, is meticulously organized and presented in detail in Tables 7, 8 and 9 and represented in Fig. 9. The efficacy of ANN-PSO-NNA approach is visualized in Fig. 8, where the convergence of the fitness function is visually highlighted across one hundred (100) independent runs. This visual representation effectively showcases the prowess of ANN-PSO-NNA hybrid methodology in steering the fitness function towards convergence.

**Table 7:** The best optimized set of weighs through ANN-PSO-NNA for z (t) for with ${\varvec{\sigma}}$ = 1, R = 2 and B = 3.

${w}_{{x}_{i}}$	${b}_{{x}_{i}}$	${a}_{{x}_{i}}$
3.503617888	3.511915862	3.02525538
− 4.393968866	8.507038958	− 7.393271149
− 2.403516491	9.966421862	9.464614611
7.854384238	3.425655586	− 5.281168576
1.703476972	− 0.953262657	1.209537784
5.236197263	− 3.358610462	− 0.116408869
− 3.049683127	− 5.807551204	1.255064755
3.631243242	− 6.040301282	− 0.894481965
6.103799032	− 7.454668307	− 0.358929329
− 7.380446107	− 2.726458661	− 3.730429637

**Table 8:** The best optimized set of weighs through ANN-PSO-NNA for y (t) for with ${\varvec{\sigma}}$ = 1, R = 2 and B = 3.

${w}_{{y}_{i}}$	${b}_{{y}_{i}}$	${a}_{{y}_{i}}$
2.411902882	9.99999947	− 4.288030635
− 0.867818886	1.344197673	1.714961449
0.528883147	5.283596026	-1.351852251
− 4.783753496	8.893206089	2.059829198
2.83734712	− 0.921885445	− 3.361964157
1.468945954	− 0.500428421	7.177176409
4.763736931	1.363274309	− 2.368845466
− 3.769246667	− 8.293914627	0.682026543
− 4.806824196	4.49947074	0.399927884
1.636435577	2.016901902	3.344084251

**Table 9:** The best optimized set of weighs through ANN-PSO-NNA for z (t) for with ${\varvec{\sigma}}$ = 1, R = 2 and B = 3.

${w}_{{z}_{i}}$	${b}_{{z}_{i}}$	${a}_{{z}_{i}}$
− 1.757509396	10.00000000	− 0.354934701
− 3.075441723	− 5.344302325	− 2.157892592
− 5.648846786	− 5.184966581	3.82154196
0.237772347	10.00000000	− 2.035385467
3.468149774	5.055246146	− 4.682569478
6.185459959	6.268931489	2.880252387
2.621306784	− 4.23911228	0.144111911
7.381804872	6.671293763	0.110750982
1.696717528	3.249311372	4.184779795
− 6.49445449	− 5.520027551	4.058179226

**Figure 10:**
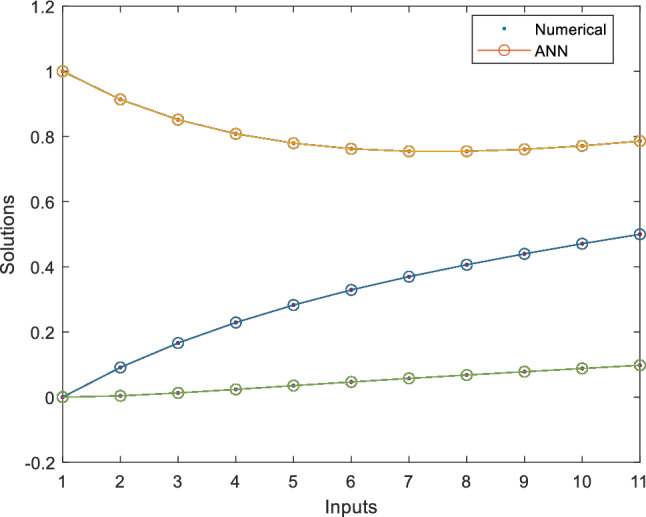
Comparison solution of Lorenz differential equation using NDsolve and ANN-PSO-NNA for case 2.

**Figure 11:**
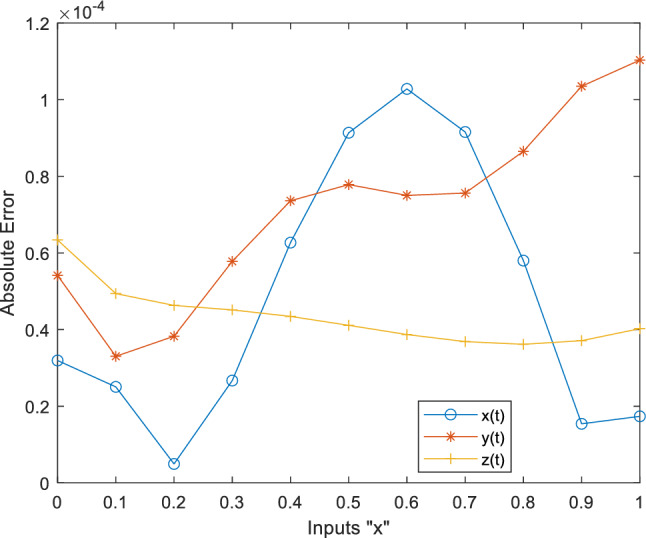
Absolute error between ANN-PSO-NNA and NDsolve for case 2.

Delving into the realm involves exploring of proposed ANN-PSO-NNA to compute and predict the ANN based $\widehat{x}\left(t\right), \widehat{y}\left(t\right)$ and $\widehat{z}\left(t\right)$. These predicted numerical values are then thoroughly tabulated in Tables 10, 11 and 12. This study takes a step further by conducting a comprehensive comparison analyze, pitting the finding from the NDsolve method against those generated by innovative numerical proposed ANN-PSO-NNA hybrid algorithm represented in Fig. 10. The absolute errors (AE) between NDsolve and ANN-PSO-NNA are tabulated in Tables 10, 11 and 12, visually represented in Fig. 11. Also focus on assessing the performance of the ANN-PSO-NNA algorithm through a comprehensive analysis of its convergence behavior. To ensure robustness, the ANN-PSO-NNA algorithm testing across a hundred (100) independent runs for fitness function.

**Table 10:** An evaluation of the accuracy of x (t) using NDsolve and ANN-PSO-NNA for case 1, with ${\varvec{\sigma}}$ = 0.1, R = 0.2 and B = 0.3.

t	${x(t)}_{Numerical}$	${\widehat{x}(t)}_{ANN}$	${AE(x(t))}_{ANN}$
0	0	7.91E−05	7.91E−05
0.1	0.090785457	0.091204098	4.19E−04
0.2	0.165933258	0.166242063	3.09E−04
0.3	0.22894329	0.2290397	9.64E−05
0.4	0.282557293	0.282709483	1.52E−04
0.5	0.32892403	0.329176282	2.52E−04
0.6	0.369729351	0.369908234	1.79E−04
0.7	0.406297807	0.406394754	9.69E−05
0.8	0.439671484	0.439867581	1.96E−04
0.9	0.470671218	0.470841364	1.70E−04
1	0.499943958	0.4998461	9.79E−05

**Table 11:** An evaluation of the accuracy of y (t) using NDsolve and ANN-PSO-NNA for case 1, with ${\varvec{\sigma}}$ = 0.1, R = 0.2 and B = 0.3.

t	${y(t)}_{Numerical}$	${\widehat{y}(t)}_{ANN}$	${AE(y(t))}_{ANN}$
0	1	0.999948246	5.18E−05
t	0.91389143	0.913523997	3.67E−04
0	0.851582403	0.851366403	2.16E−04
0.1	0.808036713	0.80814798	1.11E−04
0.2	0.779303548	0.779367483	6.39E−05
0.3	0.762305151	0.762158511	1.47E−04
0.4	0.754648985	0.754540266	1.09E−04
0.5	0.754473923	0.754615061	1.41E−04
0.6	0.76032912	0.760554328	2.25E−04
0.7	0.771080031	0.771160612	8.06E−05
0.8	0.785836227	0.785903259	6.70E−05
0.9	1	0.999948246	5.18E−05
1	0.91389143	0.913523997	3.67E−04

**Table 12:** An evaluation of the accuracy of z (t) using NDsolve and ANN-PSO-NNA for case 1, with ${\varvec{\sigma}}$ = 0.1, R = 0.2 and B = 0.3.

t	${z(t)}_{Numerical}$	${\widehat{z}(t)}_{ANN}$	${AE(z(t))}_{ANN}$
0	0	4.00E−05	3.99963E−05
0.1	0.003987329	0.004023065	3.57361E−05
0.2	0.012902208	0.012927978	2.57704E−05
0.3	0.023826656	0.023863057	3.64013E−05
0.4	0.035274975	0.035339763	6.4788E−05
0.5	0.046564683	0.046655244	9.05611E−05
0.6	0.05745071	0.057543985	9.32752E−05
0.7	0.067915471	0.067981618	6.61468E−05
0.8	0.078050717	0.07807296	2.22425E−05
0.9	0.087992822	0.087985296	7.52576E−06
1	0.097888752	0.097904612	1.58602E−05

**Figure 12:**
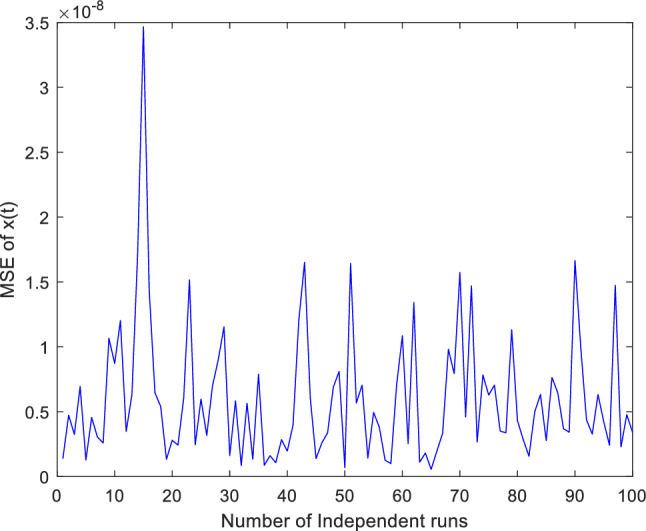
Mean square error (MSE) plotted over 100 independent runs of ${\varvec{x}}({\varvec{t}})$ for case 2.

**Figure 13:**
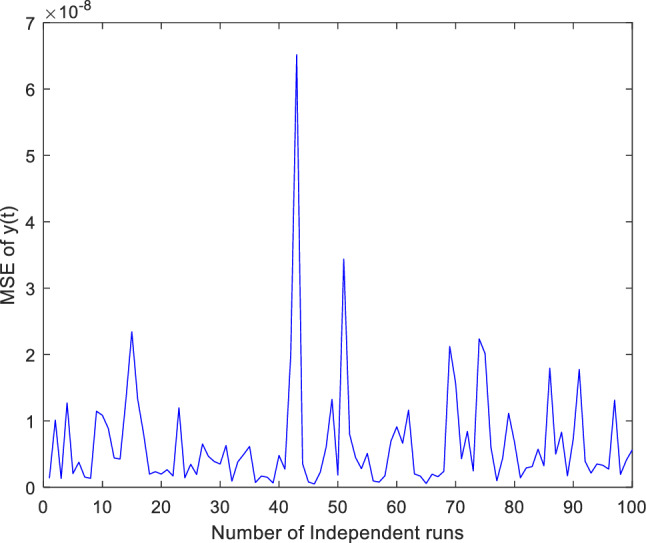
Mean square error (MSE) plotted over 100 independent runs of ${\varvec{y}}({\varvec{t}})$ for case 2.

**Figure 14:**
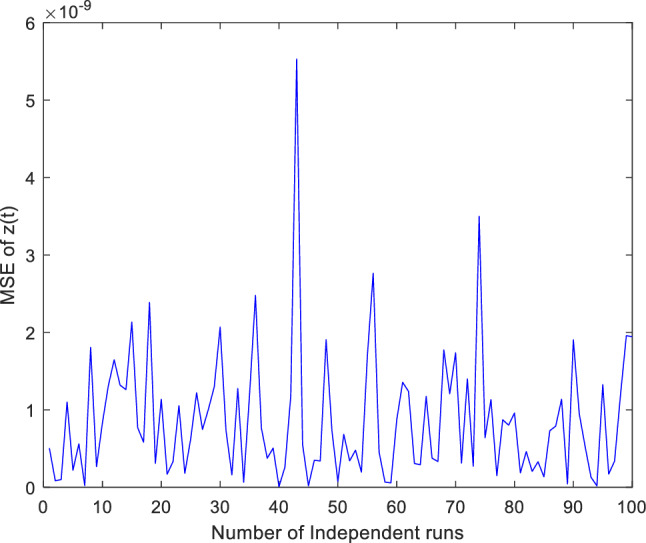
Mean square error (MSE) plotted over 100 independent runs of ${\varvec{z}}({\varvec{t}})$ for case 2.

To quantitatively evaluate its effectiveness, compute the mean square error (MSE) across one hundred (100) independence runs. The obtained numerical values of the MSE provide valuable analysis to check ability to converge towards optimal solutions of ANN-PSO-NNA. The variations in MSE across one hundred (100) independent runs are visually presented through plots offering a clear depiction of the ANN-PSO-NNA hybrid algorithm convergence trends. The plotted MSE values for different experimental scenarios are showcased in Figs. 12, 13, and 14 for case 2. To accentuate the study robustness, a comprehensive statistical analysis is conducted to draw insightful comparisons between the outcomes of two distinct methods: NDsolve and ANN. In particular, the analysis extends across an extensive set of one hundred (100) independent runs, yielding a rich array of data points for evaluation. This encompassing analysis takes into account key statistical measures encompassing minimum, maximum, average, and standard deviation values are tabulated in Table 13. These measures collectively offer a efficiency of the algorithmic performance and its consistency.

**Table 13:** Summary of absolute error between ND solve and ANN-PSO-NNA across hundred (100) independence runs for $x\left(t\right), y\left(t\right)$ and $(t)$.

	x(t) mean	x(t) min	x(t) max	x(t) STD	y(t) mean	y(t) min	y(t) max	y(t) STD	z(t) mean	z(t) min	z(t) max	z(t) STD
Case 1												
x = 0	3.132E−06	3.132E−06	8.69E−06	2.560E−06	4.19E−06	3.53E−07	1.27E−05	3.672E−06	4.167E−06	3.93E−07	2.27E−05	6.566E−06
x = 0.1	4.148E−05	4.148E−05	4.93E−05	4.069E−06	3.46E−05	2.69E−05	4.59E−05	5.32E−06	3.136E−05	1.14E−05	3.75E−05	7.342E−06
x = 0.2	4.530E−05	4.530E−05	5.24E−05	4.275E−06	1.29E−05	6.01E−06	2.44E−05	5.104E−06	3.779E−05	1.94E−05	4.35E−05	6.743E−06
x = 0.3	4.905E−06	4.905E−06	9.95E−06	2.608E−06	1.55E−05	9.27E−06	2.68E−05	4.863E−06	1.190E−05	5.14E−06	1.61E−05	3.137E−06
x = 0.4	1.933E−05	1.933E−05	2.90E−05	4.792E−06	2.82E−05	2.25E−05	3.91E−05	4.63E−06	7.365E−06	2.37E−06	2.14E−05	5.416E−06
x = 0.5	6.017E−06	6.017E−06	1.19E−05	3.248E−06	7.74E−06	2.54E−06	1.82E−05	4.414E−06	4.248E−06	6.46E−07	1.12E−05	3.367E−06
x = 0.6	5.332E−05	5.332E−05	6.10E−05	4.638E−06	4.83E−05	3.83E−05	5.31E−05	4.204E−06	2.892E−05	1.53E−05	3.46E−05	5.379E−06
x = 0.7	7.96E−05	7.96E−05	8.84E−05	4.774E−06	1.10E−04	1.01E−04	1.15E−04	4.005E−06	4.957E−05	3.46E−05	5.61E−05	5.927E−06
x = 0.8	5.327E−05	5.327E−05	6.28E−05	4.990E−06	1.50E−04	1.42E−04	1.55E−04	3.820E−06	4.563E−05	2.91E−05	5.31E−05	6.576E−06
x = 0.9	9.571E−06	9.571E−06	1.76E−05	4.899E−06	1.71E−04	1.63E−04	1.76E−04	3.648E−06	2.096E−05	3.49E−06	2.87E−05	6.910E−06
x = 1	2.646E−05	2.646E−05	3.55E−05	4.313E−06	2.27E−04	2.20E−04	2.32E−04	3.489E−06	1.218E−05	5.82E−06	1.79E−05	3.508E−06
Case 2												
x = 0	6.99E−05	1.75E−06	1.53E−04	4.70E−05	4.10E−05	1.07E−07	1.19E−04	3.51E−05	3.29E−05	1.62E−06	7.61E−05	2.36E−05
x = 0.1	6.69E−05	3.12E−05	1.39E−04	3.67E−05	3.04E−05	4.08E−07	8.25E−05	2.32E−05	3.03E−05	3.91E−06	6.45E−05	1.91E−05
x = 0.2	7.24E−05	4.67E−05	1.39E−04	2.99E−05	2.18E−05	1.06E−06	5.20E−05	1.76E−05	2.81E−05	4.04E−06	5.52E−05	1.60E−05
x = 0.3	7.76E−05	4.58E−05	1.38E−04	2.81E−05	2.48E−05	1.48E−06	6.59E−05	2.03E−05	2.64E−05	4.23E−06	4.83E−05	1.40E−05
x = 0.4	8.35E−05	3.66E−05	1.35E−04	2.92E−05	3.58E−05	4.37E−06	8.17E−05	2.63E−05	2.52E−05	4.69E−06	4.33E−05	1.29E−05
x = 0.5	9.20E−05	4.38E−05	1.35E−04	3.12E−05	5.04E−05	5.76E−06	1.01E−04	3.04E−05	2.46E−05	5.57E−06	4.15E−05	1.21E−05
x = 0.6	1.01E−04	5.73E−05	1.61E−04	3.31E−05	6.82E−05	2.89E−05	1.25E−04	3.11E−05	2.46E−05	7.02E−06	4.28E−05	1.16E−05
x = 0.7	1.01E−04	4.89E−05	1.72E−04	3.43E−05	8.69E−05	5.53E−05	1.54E−04	3.28E−05	2.51E−05	9.15E−06	4.41E−05	1.12E−05
x = 0.8	8.05E−05	4.08E−05	1.52E−04	3.21E−05	1.02E−04	6.96E−05	1.77E−04	3.67E−05	2.63E−05	1.21E−05	4.54E−05	1.09E−05
x = 0.9	4.20E−05	6.04E−06	1.03E−04	2.84E−05	1.06E−04	6.43E−05	1.85E−04	3.97E−05	2.80E−05	1.59E−05	4.68E−05	1.07E−05
x = 1	3.72E−05	1.76E−05	7.84E−05	2.10E−05	9.78E−05	5.50E−05	1.66E−04	3.79E−05	3.03E−05	1.97E−05	4.84E−05	1.08E−05

**Table:** 

Case 1:	Case 2:
${\widehat{x}(t)}_{ANN-PSO-NNA}=\frac{-0.813325309}{{1+e}^{(0.124153837{\text{t}}-1.977661201)}}+\frac{-3.905335359}{{1+e}^{(-7.842802359{\text{t}}-7.010510834)}}+\frac{2.070647136}{{1+e}^{(-1.13823337{\text{t}}-1.072293243)}}+\frac{-2.459776325}{{1+e}^{*(-2.87089813{\text{t}}-0.653293577)}}+\frac{0.020306929}{{1+e}^{(0.390174591{\text{t}}+4.893449037)}}+\frac{0.59295416}{{1+e}^{(-1.651579931{\text{t}}-3.117918392)}}+\frac{-1.311168679}{{1+e}^{(1.540327448{\text{t}}-0.272077258)}}+\frac{0.239241627}{{1+e}^{(1.778650834{\text{t}}+0.575686377)}}+\frac{-1.76626202}{{1+e}^{(0.591203789{\text{t}}-1.30910321)}}+\frac{2.260099588}{{1+e}^{(0.751550886{\text{t}}-0.727266222)}}$	${\widehat{x}\left(t\right)}_{ANN-PSO-NNA}=\frac{3.02525538}{1+{e}^{-(3.503617888{\text{x}}+3.511915862)}}+\frac{-7.393271149}{1+{e}^{-(-4.393968866{\text{x}}+8.507038958)}}+\frac{9.464614611}{1+{e}^{-(-2.403516491{\text{x}}+9.966421862)}}+\frac{-5.281168576}{1+{e}^{-(7.854384238{\text{x}}+3.425655586)}}+\frac{1.209537784}{1+{e}^{-(1.703476972{\text{x}}-0.953262657)}}+\frac{-0.116408869}{1+{e}^{-(5.236197263{\text{x}}-3.358610462)}}+\frac{1.255064755}{1+{e}^{-(-3.049683127{\text{x}}-5.807551204)}}+\frac{-0.894481965}{1+{e}^{-(3.631243242{\text{x}}-6.040301282)}}+\frac{-0.358929329}{1+{e}^{-(6.103799032-7.454668307)}}+\frac{-3.730429637}{1+{e}^{-(-7.380446107-2.726458661)}}$
${\widehat{y}(t)}_{ANN-PSO-NNA}=\frac{1.79513648}{{1+e}^{(-1.619284162{\text{t}}+2.33332237)}}+\frac{-3.479169522}{{1+e}^{(1.049353281{\text{t}}+0.391080791)}}+\frac{1.901550548}{{1+e}^{(-1.868873265{\text{t}}-2.946745976)}}+\frac{2.405750129}{{1+e}^{(-5.427098595{\text{t}}+1.388433611)}}+\frac{0.791306332}{{1+e}^{(-2.256709224{\text{t}}+1.136508252)}}+\frac{1.568062177}{{1+e}^{(-3.343812861{\text{t}}-1.295923739)}}+\frac{-3.497286829}{{1+e}^{(-8.177244791{\text{t}}-0.800059922)}}+\frac{3.55394297}{{1+e}^{(1.339271967{\text{t}}-1.199927409)}}+\frac{2.043766309}{{1+e}^{(2.527430403{\text{t}}-1.014961309)}}+\frac{0.322003823}{{1+e}^{(1.493407826{\text{t}}+3.11031704)}}$	${\widehat{y}(t)}_{ANN-PSO-NNA}=\frac{-4.288030635}{1+{e}^{-(2.411902882{\text{t}}+9.99999947)}}+\frac{1.714961449}{1+{e}^{-(-0.867818886{\text{t}}+1.344197673)}}+\frac{-1.351852251}{1+{e}^{-(0.528883147{\text{t}}+5.283596026)}}+\frac{2.059829198}{1+{e}^{-(-4.783753496{\text{t}}+8.893206089)}}+\frac{-3.361964157}{1+{e}^{-(2.83734712{\text{t}}-0.921885445)}}+\frac{7.177176409}{1+{e}^{-(1.468945954{\text{t}}-0.500428421)}}+\frac{-2.368845466}{1+{e}^{-(4.763736931{\text{t}}+1.363274309)}}+\frac{0.682026543}{1+{e}^{-(-3.769246667{\text{t}}-8.293914627)}}+\frac{0.399927884}{1+{e}^{-(-4.806824196{\text{t}}+4.49947074)}}+\frac{3.344084251}{1+{e}^{-(1.636435577{\text{t}}+2.016901902)}}$
${\widehat{z}(t)}_{ANN-PSO-NNA}=\frac{1.640553936}{{1+e}^{(-2.655547759{\text{t}}-0.646854678)}}+\frac{-1.030352672}{{1+e}^{(-2.065588875{\text{t}}+3.135717185)}}+\frac{2.323983541}{{1+e}^{(1.302367848{\text{t}}+0.998378559)}}+\frac{-2.313212076}{{1+e}^{(-0.642094131{\text{t}}-0.391409694)}}+\frac{0.750563972}{{1+e}^{(0.343277015{\text{t}}+1.435755696)}}+\frac{-1.072402348}{{1+e}^{(1.233927562{\text{t}}-0.587671128)}}+\frac{1.077467937}{{1+e}^{(0.639052951{\text{t}}-0.168720266)}}+\frac{-0.725073923}{{1+e}^{(1.769849572{\text{t}}-0.426233397)}}+\frac{2.009219372}{{1+e}^{(0.773859075{\text{t}}-1.259451854)}}+\frac{9.576093207}{{1+e}^{(0.596666302{\text{t}}-0.013176696)}}$	${\widehat{z}(t)}_{ANN-PSO-NNA}=\frac{-0.354934701}{1+{e}^{-(-1.757509396{\text{t}}+10.0000000)}}+\frac{-2.157892592}{1+{e}^{-(-3.075441723{\text{t}}-5.344302325)}}+\frac{3.82154196}{1+{e}^{-(-5.648846786{\text{t}}-5.184966581)}}+\frac{-2.035385467}{1+{e}^{-(0.237772347{\text{t}}+10.0000000)}}+\frac{-4.682569478}{1+{e}^{-(3.468149774{\text{t}}+5.055246146)}}+\frac{2.880252387}{1+{e}^{-(6.185459959{\text{t}}+6.268931489)}}+\frac{0.144111911}{1+{e}^{-(2.621306784{\text{t}}-4.23911228)}}+\frac{0.110750982}{1+{e}^{-(7.381804872{\text{t}}+6.671293763)}}+\frac{4.184779795}{1+{e}^{-(1.696717528{\text{t}}+3.249311372)}}+\frac{4.058179226}{1+{e}^{-(-6.49445449-5.520027551)}}$

## Conclusion

In conclusion, the rapid evolution of unsupervised artificial neural networks (ANN) technique has opened up exciting methodologies for addressing complex nonlinear differential equation problems in various engineering domains. This study has analyzed on the power of machine learning algorithm to solve the non-linear Lorenz differential equation, using a novel approach of artificial neural networks (ANN) combining with particle swarm optimization (PSO) hybrid with neural networks algorithm (NNA). The Lorenz differential equations renowned for the chaotic behavior, have served as a fundamental benchmark for scientific exploration.

Using the ANN-PSO-NNA hybrid approach, our objective has been to enhance effectiveness, validation and accuracy of solving ANN based Lorenz differential equation, enabling more accurate approximation of problems. Further for evaluated the ANN-PSO-NNA through a comprehensive statistical analysis involving one hundred (100) independent runs. The metrics for the statistical analysis such as minimum, maximum, standard deviation, average, and mean square error values between the NDsolve and ANN-PSO-NNA. The ANN based fitness function optimized through hybrid PSO-NNA algorithms for minimum error achieved of $x\left(t\right), y\left(t\right)$ and $z\left(t\right)$ upto $1.75\times {10}^{-06}, 1.07\times {10}^{-07}$ and $3.93\times {10}^{-07}$ respectivily, for the highly nonlinear chaotic system the PSO-NNA maybe achieve less accuracy and use the more computational cost, to tackle these types of system will be used quantum based optimization algorithms. In the future, enhancing the accuracy and efficiency of solving non-linear dynamics problems will be a priority using other heuristic optimization algorithms, such as genetic algorithms (GA), ant colony optimization (ACO), firefly algorithm (FA) and quantum computing based algorithm.

## Data Availability

The data used and/or analyzed during the current study available from the corresponding author on reasonable request.
